# A Multi-Gradient-Descent-Integrated Physics-Informed Autoencoder for Sensor Fault Detection in Data Center Chillers

**DOI:** 10.3390/s26103025

**Published:** 2026-05-11

**Authors:** Xinyue Shen, Pan Li, Chen Xu, Chengliang Xu

**Affiliations:** School of Urban Construction, Wuhan University of Science and Technology, Wuhan 430065, China; shenxinyue118@wust.edu.cn (X.S.);

**Keywords:** data center, chiller, sensor fault detection, physics-informed autoencoder, multiple gradient descent algorithm

## Abstract

The operating status of chillers in data centers critically affects temperature stability and energy efficiency, imposing stringent requirements on the accuracy, robustness, and adaptability of sensor fault detection systems. Existing Physics-Informed Autoencoders (PIAE) for chiller fault detection face challenges such as gradient conflicts and limited generalization, especially under complex fault scenarios like sensor drift, stuck faults, and precision attenuation. To address these limitations, we propose a Multi-Gradient Descent Algorithm-integrated PIAE (MGDA-PIAE), which embeds the chiller thermal balance equation as a hard constraint and dynamically determines Pareto-optimal weights between data reconstruction and physical consistency. By unifying feature learning with physical law constraints, the model achieves improved generalization and stable performance across diverse operational conditions. Validation using operational data from a data center in Guangzhou demonstrates that MGDA-PIAE significantly outperforms conventional AE and PIAE models: the average recall increases by approximately 20% and the F1-score by 10%, while for flow sensor faults, the F1-score improves by over 80% compared with AE and 20% compared with PIAE. Further tests on multiple sensor types confirm that the model maintains high-precision detection with low false negatives under varying operating modes. By adjusting a single physical threshold, MGDA-PIAE can flexibly meet different fault detection requirements, providing a practical and reliable solution for maintaining the efficient, stable, and safe operation of data center refrigeration systems.

## 1. Introduction

Sensors are indispensable components in heating, ventilation, and air conditioning (HVAC) systems, serving as the primary data acquisition elements that enable real-time monitoring and control of indoor environmental conditions. The healthy operation of these sensors lays a crucial foundation for implementing advanced control strategies aimed at improving building energy efficiency and indoor environmental quality [1,2,3]. Buildings account for a substantial portion of global energy consumption—approximately 30–40% of total primary energy use—and within commercial buildings, HVAC systems are the dominant energy end-use, representing about 40–50% of total energy consumption [4,5]. This significant energy footprint underscores the urgency of optimizing HVAC operations to meet global energy efficiency and carbon neutrality targets. Achieving these goals relies heavily on the efficient operation of HVAC systems, which in turn depends on sophisticated control strategies that leverage real-time, accurate sensor data [6]. These strategies include demand-controlled ventilation, optimal start-stop scheduling, model predictive control, and fault-adaptive controls, all of which require reliable sensor measurements to function effectively.

However, in real-world environments, sensors in HVAC systems are susceptible to various faults due to harsh operating conditions such as temperature extremes, humidity, dust accumulation, and vibration, as well as inadequate maintenance and sensor aging over time [7,8,9]. Common sensor faults include bias (constant offset), drift (gradual change over time), precision degradation (increased noise), and complete failure (stuck or out-of-range values). These faults not only significantly impact the system operation and degrade control loop performance but also exacerbate energy waste and undermine system reliability and occupant comfort [10,11,12]. Research has demonstrated that sensor faults can lead to an additional 15–30% increase in energy consumption due to suboptimal control actions [13]. Moreover, sensor inaccuracies can severely affect downstream tasks that rely on sensor data, such as fault detection and diagnosis for other components. For instance, studies show that thermal fault diagnosis accuracy may drop by 13.7–53.4% when input sensor data are corrupted [8]. Therefore, timely and accurate detection of sensor faults is essential for maintaining the reliable, efficient, and sustainable operation of modern HVAC systems, particularly in large and complex facilities such as data centers, hospitals, and commercial complexes, where the number of sensors is substantial and fault scenarios are intricate [14,15,16].

To address this challenge, the academic community has developed various Fault Detection and Diagnosis (FDD) methods for HVAC systems. These methods can be broadly categorized into three types: rule-based, physics-based, and data-driven approaches [2,17]. Rule-based methods rely on expert-defined thresholds or fixed logical rules to detect anomalies. They are simple to implement and computationally efficient but lack quantitative analysis capabilities and struggle to adapt to diverse operating conditions or evolving system behavior over time [18]. Physics-based methods, on the other hand, are grounded in fundamental physical laws such as energy and mass conservation, thermodynamics, and fluid mechanics. They offer strong interpretability and high accuracy when the underlying models are well-calibrated. However, their reliance on precise mathematical models makes their development complex and costly, requiring detailed system knowledge and extensive calibration efforts. Furthermore, their accuracy tends to degrade over time due to equipment aging, wear and tear, and changes in operating conditions, making recalibration difficult and expensive [10].

To overcome the limitations of rule-based and physics-based approaches, data-driven methods have emerged as a vibrant research area. These techniques utilize statistical analysis and machine learning algorithms to learn patterns of normal and faulty system behavior directly from historical operational data, without requiring explicit physical models [19]. Among these, Principal Component Analysis (PCA) and its variants are widely used for multi-sensor fault detection due to their dimensionality reduction capabilities, which help manage high-dimensional sensor data [20,21]. PCA projects the original sensor measurements onto a lower-dimensional subspace that captures the majority of variance, and faults are detected when the residuals exceed a threshold. However, PCA is based on linear assumptions and thus struggles to capture the complex nonlinear relationships that are inherent in HVAC sensor data, such as those arising from nonlinear physical processes or control actions. Additionally, the dimensionality reduction process may discard information that is critical for identifying minor or incipient faults, leading to missed detections [22,23]. Support Vector Machines (SVM) offer a way to handle nonlinearity through kernel functions, effectively mapping input data into a higher-dimensional feature space where linear separation is possible. SVM has been successfully applied to chiller fault detection [24,25]. However, in large-scale, high-dimensional systems, the selection of appropriate kernel functions and hyperparameters is complex and often requires extensive cross-validation. Moreover, the computational cost of training and deploying SVM models can be prohibitive when dealing with thousands of sensors and high-frequency data streams [24].

In recent years, deep learning methods, particularly autoencoders (AE) and various neural network architectures, have gained significant attention for their ability to learn complex nonlinear features directly from raw sensor data [26,27,28]. Autoencoders, which consist of an encoder that compresses input data into a latent representation and a decoder that reconstructs the original input, can capture intricate dependencies and patterns without requiring explicit fault labels, although they inherently rely on a screened nominal dataset to establish a healthy baseline. Variants such as variational autoencoders [29], denoising autoencoders [16], and long short-term memory networks [30] have been explored for time-series sensor data. Despite their strong representational power, deep models suffer from a “black-box” nature, which undermines their interpretability and reliability in safety-critical applications [31,32]. Understanding why a model flags a particular sensor reading as faulty is crucial for building trust and enabling corrective actions. Furthermore, in scenarios involving simultaneous faults in multiple sensors, these methods often require large amounts of labeled training data covering diverse fault types and severities, which is rarely available in practice. The computational resources needed for training and inference can also be substantial, limiting real-time deployment [33,34].

In summary, the existing data-driven methods still face three major challenges when applied to large, complex HVAC systems: (1) potential information loss during nonlinear processing and feature extraction, which may compromise the detection of subtle or early-stage faults; (2) the computational burden imposed by high-dimensional sensor data, which hinders scalability and real-time performance; and (3) the lack of reliability and interpretability that is inherent in purely data-driven approaches, particularly when it comes to detecting simultaneous multi-sensor faults where interactions between faults complicate the detection task. These challenges motivate the need for novel approaches that combine the strengths of data-driven learning with domain knowledge to enhance robustness, interpretability, and scalability.

To address these problems, this paper proposes a novel physics-guided deep learning method, the Multi-Gradient Descent Algorithm-based Physics-Informed Autoencoder (MGDA-PIAE)—for sensor fault detection in large-scale, complex HVAC systems. Inspired by the emerging field of physics-informed neural networks, this method integrates the system’s thermodynamic principles directly into the autoencoder architecture. Specifically, we embed physical laws, such as energy balance equations and heat transfer relationships, into the loss function as soft constraints. By customizing the loss function to penalize predictions that violate these physical laws, the model learns to balance data reconstruction accuracy with physical consistency, achieving robust and interpretable feature learning. The training process employs the Multi-Gradient Descent Algorithm (MGDA), an optimization strategy that dynamically adjusts the trade-off between statistical accuracy (minimizing reconstruction error) and physical plausibility (minimizing physical constraint violations). MGDA seeks a Pareto optimal solution where neither objective can be improved without degrading the other, effectively overcoming the limitations of purely data-driven methods in complex multi-fault scenarios where data alone may be insufficient.

The experiment used real operational data from the chill water room of a large data center in Guangzhou, which includes multiple chillers, pumps, cooling towers, and hundreds of sensors measuring temperatures, pressures, flow rates, and power consumption. The data covers three years of normal operation. On this basis, we artificially injected faults (single and simultaneous) into the test data to evaluate the detection performance. We comprehensively compared the MGDA-PIAE against traditional baseline methods, including the vanilla Autoencoder (AE) and the standard Physics-Informed Autoencoder (PIAE).

The main contributions and core innovations of this work are summarized as follows:

(1) We propose a Multi-Gradient Descent Algorithm-based Physics-Informed Autoencoder (MGDA-PIAE), which fundamentally addresses the gradient conflict between the data reconstruction and physical constraint objectives that are inherent in conventional PIAE models through dynamic Pareto-optimal optimization.
(2) The proposed model achieves a significant performance improvement in detecting faults of core flow sensors in data center chillers. Specifically, compared with conventional AE and PIAE models, the F1-score increases by 80% and 20%, respectively, with particularly notable gains in identifying moderate deviation faults.
(3) Moreover, the model allows the flexible adjustment of a single physical constraint threshold to switch between different operational modes. This capability enables practitioners to prioritize either detection sensitivity or physical feasibility depending on application requirements—for instance, enforcing stricter physical consistency in critical environments such as cleanrooms, while favoring higher detection sensitivity in energy-centric settings such as data centers—thereby enhancing its practical deployment flexibility.

## 2. Methodology

### 2.1. Overview

**Figure 1:**
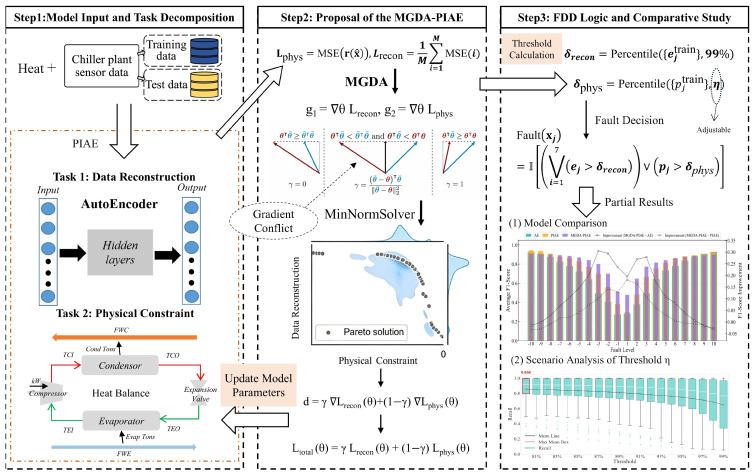
Overall design of the MGDA-PIAE model.

The Physics-Constrained Autoencoder Fault Detection Model based on Multi-Objective Optimization Gradient Descent (hereinafter referred to as the MGDA-PIAE model) solves the Pareto optimal solution for the data reconstruction loss and physical constraint loss through the Multiple Gradient Descent Algorithm, realizing the dynamic balance of their weights during the training process. This addresses the mismatch between the data fitting accuracy and physical consistency in traditional single-objective models, and improves the comprehensive performance and system generalization ability of fault detection. The overall architecture of the model is shown in Figure 1. After the sensor data is input, the process is as follows:

Step 1: Model Input and Task Decomposition.

The preprocessed Chiller plant sensor data is divided into training and test datasets, and then decomposed into two parallel core tasks.

Data reconstruction task: Sensor data first passes through the “shared encoder–latent space–shared decoder” pipeline to complete encoding and decoding, after which the reconstruction loss is calculated to quantify the data fitting accuracy.

Physical constraint task: The output of the shared decoder is transmitted to the thermal equilibrium model, and the physical loss is computed based on the energy conservation mechanism to ensure that the model output conforms to thermodynamic laws.

Step 2: MGDA-PIAE Model Optimization.

The reconstruction loss and physical loss from the two tasks are input into the MGDA optimization module. The MinNormSolver is used to solve the Pareto-optimal weights (specifically, the shared parameter γ) for the dual tasks, addressing the gradient conflict between them. A joint gradient is then calculated to update the model’s shared parameters, and an adjustable physical error threshold is derived from the optimal weights.

Step 3: Fault Detection and Result Analysis.

By combining the fixed reconstruction error threshold (99th percentile) and the adjustable physical error threshold, a joint fault decision is made through the “logical OR” rule. The final fault detection results are output, and a further model performance comparison and scenario analysis of the threshold η are conducted.

The specific implementation process is detailed in the subsequent sections.

### 2.2. Principle and Construction of the MGDA-PIAE Model

#### 2.2.1. Multi-Objective Optimization and Pareto Optimality Theory in FDD

The fault detection task based on deep learning can be regarded as a multi-objective optimization problem [35], where the data reconstruction task and the physical constraints existing in the data are the two objectives to be optimized. If a simple fixed weight weighted sum strategy is adopted, the optimization process is likely to be dominated by a single task, making the performance of the other task unable to be fully optimized, and thus difficult to converge to a good balance point—this is a typical manifestation of the aforementioned gradient conflict problem. Its multi-objective optimization framework can be expressed as follows:

(1) $$\underset{\theta \in \Theta }{\min}{\left[L_{\mathrm{recon}}(\theta ),\;L_{\mathrm{phys}}(\theta )\right]}^{T}$$

where θ is the model parameter, *L*_recon_ (θ) is the loss function quantifying the accuracy of data reconstruction, and *L*_phys_ (θ) is the loss function quantifying the degree to which the model output violates known physical laws.

Under this framework, the underlying representation layer of the model can be shared through multi-task learning to achieve knowledge transfer and mutual regularization between the data reconstruction task and the physical constraint task [36]. Specifically:

(1) The data reconstruction task tends to learn the compact statistical representation of sensor data, is highly sensitive to local subtle deviations, and aims to accurately recover the original input data to capture abnormal patterns at the sensor level;
(2) The physical constraint task forces the latent variable representation of the model to comply with the macroscopic laws of thermodynamics and emphasizes the global consistency of the overall energy balance, to ensure that the model output conforms to the first principles and to improve the detection ability of system-level faults.

The optimization gradients of these two tasks may have directional conflicts in the parameter space, which is manifested in that the gradient descent direction of one task may be the ascent direction for the other task.

The solution to multi-objective optimization is a set of Pareto optimal solutions, forming the Pareto Frontier. At any point on this frontier (corresponding to a set of model parameters θ), it is impossible to improve any objective without deteriorating other objectives, which is exactly in line with the engineering requirement of finding the best balance between data fitting accuracy and compliance with physical laws in HVAC fault detection. Let w′ and w″ be two different solutions; if w′ is Pareto superior to w″, then it satisfies the following:

(2) $$\forall i\;\in \;\left\{\mathrm{recon},\mathrm{phys}\right\}:L_{i}\left(w^{\prime }\right)\;\leq \;L_{i}(w^{\prime \prime })\;\mathrm{and}\;\exists j:L_{j}(w^{\prime })\;<{\;L}_{j}(w^{\prime \prime })$$

**Figure 2:**
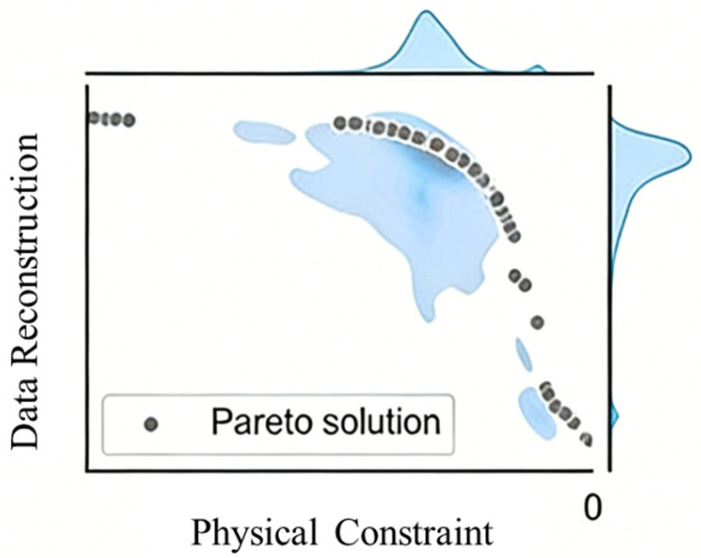
Schematic diagram of the Pareto stationary point.

In the weight feasible region (two-dimensional simplex *S* = {*w* ∈ R^2^: *w_i_* ≥ 0, ∑*w_i_* = 1}), if there exists a weight *w*^∗^ such that the gradient of the total loss *L_total_* = *w*_1_ × *L_recon_* + *w*_2_ × *L_phys_* is zero, this solution is called a Pareto Stationary Point. The relevant schematic diagram is as shown in Figure 2.

#### 2.2.2. Fusion Principle of the MGDA-PIAE Model

Based on the Pareto optimality theory, the Multiple Gradient Descent Algorithm (MGDA) can adaptively solve the optimal weights of the dual tasks to alleviate gradient conflicts. Embedding it into the Physics-Informed Autoencoder (PIAE) framework forms the MGDA-PIAE fault detection model proposed in this study, whose core principles are as follows.

##### PIAE Framework

PIAE is a fault diagnosis method that combines physical laws with the reconstruction capability of autoencoders, and it has become relatively mature in recent years. In this study, we refer to the relevant work of Ren et al. [1] to build a traditional PIAE model as a comparison model. This model integrates physical constraints with data-driven autoencoders to achieve data reconstruction and physical consistency verification during fault detection.

##### Principle of the Multiple Gradient Descent Algorithm (MGDA)

The Multiple Gradient Descent Algorithm (MGDA) was first proposed by Désidéri [37]. Sener and Koltun (2018) introduced it into the field of multi-task learning and proposed a more computationally efficient upper bound version (MGDA-UB) [38]. The algorithm and implementation described below are all based on the MGDA-UB framework. The core idea of this algorithm is to provide a Pareto optimal gradient update direction for the shared parameter layer through geometric methods, instead of relying on manually set static weights.

MGDA formulates the multi-task trade-off as a quadratic programming problem. In each iteration, the optimal gradient $g^{*}$ for shared parameters is obtained by solving the following:

(3) $$\underset{w\in R^{T}}{\min}{∥\sum_{i=1}^{T}w_{i}\nabla_{\theta }L_{i}(\theta )∥}_{2}^{2}s.t.\sum_{i=1}^{T}w_{i}\;=\;1,{\;w}_{i}\;\geq \;0$$

where **g_i_** = **∇_θ_L_i_** represents the gradient vector of the i-th task with respect to the shared parameter θ, and T = 2 corresponds to the dual-task scenario in this study. The geometric meaning of this problem is to find the point closest to the origin in Euclidean distance in the convex hull formed by all task gradients {**g_1_**, **g_2_**}. The coordinate **w_i_** of this point is the optimal weight, and the corresponding vector $g^{*}=\sum_{i=1}^{T}w_{i}\cdot g_{i}$ gives the ideal parameter update direction, which is the minimum norm gradient.

If ∥g*∥_2_ = 0, the current parameters have reached the Pareto stationary point; otherwise, the negative direction −g* is a strict common descent direction, and updating the parameters along this direction can reduce the loss values of all tasks simultaneously.

**Figure 3:**
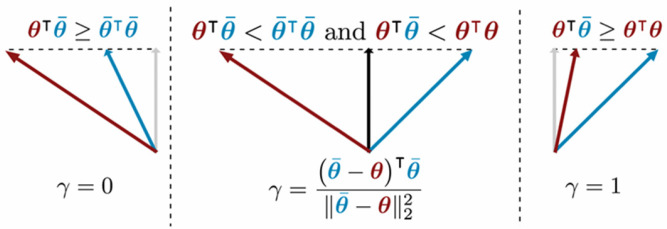
Schematic diagram of gradient descent (two tasks).

From an intuitive understanding of the gradient space, the Pareto optimality condition is equivalent to finding an optimal update direction for the shared parameters such that the angle between this direction and all task gradients is less than 90°. As shown in Figure 3, when the angle between the two task gradients is an acute angle, there is a clear common descent direction; when the angle is an obtuse angle, it is necessary to find a “compromise” optimal direction, such that when updating along this direction, the losses of the two tasks cannot be maximally reduced at the same time, but both can obtain acceptable improvements.

For the special case of two tasks (T = 2), this optimization problem has a closed-form solution. Let **g_1_** = ∇*L_recon_* (*θ*) and **g**_2_ = ∇*L_phys_* (*θ*); then, the calculation of the optimal weight **γ** (corresponding to **w_1_**) is divided into three cases:

(4) $$\left\{\begin{array}{cc} g_{1}^{⊤}g_{2}\;\geq \;g_{2}^{⊤}g_{2}, & \gamma \;=\;0\\ g_{1}^{⊤}g_{2}\;<\;g_{2}^{⊤}g_{2}\;\mathrm{and}\;g_{1}^{⊤}g_{2}\;<\;g_{1}^{⊤}g_{1}, & \gamma =\frac{{\left(g_{2}\;-\;g_{1}\right)}^{⊤}g_{2}}{∥g_{2}-g_{1}{∥}_{2}^{2}}\\ g_{1}^{⊤}g_{2}\;\geq {\;g}_{1}^{⊤}g_{1}, & \gamma \;=\;1 \end{array}\right.$$

These cases correspond to: (1) physical constraint task dominance (γ = 0), (2) inter-task compromise, and (3) data reconstruction task dominance (γ = 1). The final update direction is $d\;=\;\gamma \nabla L_{\mathrm{recon}}(\theta )\;+\;(1\;-\;\gamma )\nabla L_{\mathrm{phys}}(\theta )$, which ensures that the model parameters are updated in the direction of Pareto improvement in each iteration.

##### Principle of PIAE Based on MGDA

The Physics-Informed Autoencoder based on MGDA (MGDA-PIAE) addresses gradient conflicts in dual-task learning, improving model robustness and generalization. Its key idea is to embed MGDA into the PIAE framework, automatically determining Pareto-optimal weights to guide the shared encoder and decoder to learn features that align with both data statistics and physical laws.

(1) Shared parameter layer: The encoder and decoder are shared by the data reconstruction and physical constraint tasks, ensuring feature consistency and complementarity.
(2) Decoupled task loss: $L_{\mathrm{recon}}$ measures reconstruction error; $L_{\mathrm{phys}}$ measures the residual of the physical equation.
(3) MGDA dynamic weighting: Pareto-optimal weights (γ,1 − γ) balance $L_{\mathrm{recon}}$ and $L_{\mathrm{phys}}$ through quadratic programming.
(4) Synergistic gradient update: Weighted gradients define the update direction, advancing the model toward a Pareto frontier of simultaneous data fitting and physical consistency.

The differences between MGDA-PIAE and the traditional PIAE are shown in Table 1.

#### 2.2.3. Design and Implementation of Dynamic Loss Function Optimization

To achieve effective training and optimization of the model, this study first constructs a dual-task adaptive loss function framework. Based on this framework, dynamic weight optimization and the overall algorithm implementation are completed in conjunction with the Multi-Gradient Descent Algorithm (MGDA). The key design details are as follows.

##### Construction of the Loss Function

In this study, a dataset $\{X_{i}{\}}_{i=1}^{T}$ is constructed based on time-series sensor data under normal operating conditions of the chiller system, where each sample $x_{i}\;\in \;R^{7}$ represents a feature vector containing seven key variables. The physical constraint loss, data reconstruction loss, and the total loss of MGDA-PIAE are constructed. The core definitions of each loss function are as follows:

Physical constraint loss: The physical residual function $r(\hat{x})$ is defined based on the thermal balance equation of the chiller system, quantifying the degree of deviation of the system’s state from the physical laws. A small value $\epsilon \;=\;10^{-12}$ is introduced to avoid gradient explosion due to division by zero. The Mean Squared Error (MSE) is used as the computation metric for the physical constraint loss, as shown by the following:

(5) $$L_{\mathrm{phys}}\;=\;\mathrm{MSE}(r(\hat{x}))$$

Data reconstruction loss: The model is trained exclusively on the normal dataset, which leads to significant reconstruction errors when processing fault data. The MSE is again used to calculate the reconstruction error for a single sample and the batch average reconstruction loss. The final batch average data reconstruction loss is formulated as follows:

(6) $$L_{\mathrm{recon}}\;=\;\frac{1}{M}\sum_{i=1}^{M}\mathrm{MSE}(i)$$

MGDA-PIAE total loss: Traditional fixed-weight weighted sums are abandoned in favor of a dynamic, adaptive total loss constructed using the Pareto optimal weights solved online by MGDA. The formula is as follows:

(7) $$L_{\mathrm{total}}(\theta )\;=\;\gamma \cdot L_{\mathrm{recon}}(\theta )\;+\;(1\;-\;\gamma )\cdot L_{\mathrm{phys}}(\theta )$$

Here, γ ∈ [0, 1] is the Pareto optimal weight solved by MGDA. No manual parameter tuning is required, as the weight is adjusted in real time with each model parameter update. When γ approaches one, the model focuses more on the data reconstruction task, and when γ approaches zero, it focuses on the physical constraint task. This enables an adaptive balance between the data fitting capability and physical consistency.

##### Model Training Process

The core of the MGDA-PIAE model training is the collaborative optimization of the dual-task gradients using MGDA. The entire process integrates gradient computation, normalization, optimal weight solving, and parameter updates, as shown in Algorithm 1. The specific steps are as follows.

Initialization: The training and validation datasets are loaded, and the encoder and decoder parameters are initialized. The Adam optimizer is set with an initial learning rate of $5\;\times \;10^{-4}$, a batch size between 32 and 128, and other hyperparameters.

Forward propagation: The 7-dimensional sensor data is input into the model. After being mapped to a 64-dimensional latent vector by the encoder, the data is reconstructed to a 7-dimensional output by the decoder. The reconstructed data is then passed to the physical constraint module to calculate the residuals of the thermal balance equation.

Dual-task loss and gradient computation: The data reconstruction loss $L_{\mathrm{recon}}$ and the physical constraint loss $L_{\mathrm{phys}}$ are computed separately. The gradients of both losses with respect to the shared parameters are then calculated through backpropagation: $g_{1}\;=\;\nabla_{\theta }L_{\mathrm{recon}}$ and $g_{2}\;={\;\nabla }_{\theta }L_{\mathrm{phys}}$.

Gradient normalization: The ‘loss + L2 norm’ normalization strategy is employed to eliminate the optimization bias caused by the disparity in gradient magnitudes between the two tasks. The formulas for the normalized gradients are as follows:

(8) $$g_{1}^{\prime }\;=\;\frac{g_{1}}{∥L_{\mathrm{recon}}∥+∥g_{1}∥},\;g_{2}^{\prime }\;=\;\frac{g_{2}}{∥L_{\mathrm{phys}}∥+∥g_{2}∥}$$

Weight solving and parameter update: The MinNormSolver is used to solve the quadratic programming problem, yielding the Pareto optimal weight γ and the joint gradient $g^{*}\;=\;\gamma \cdot g_{1}^{\prime }\;+\;(1\;-\;\gamma )\cdot g_{2}^{\prime }$. The shared parameters are updated in the direction of the following negative joint gradient:

(9) $$\theta \;=\;\theta \;-\;\mathrm{lr}\cdot g^{*}$$

where lr is the learning rate, completing one iteration.

Early stopping strategy: The F1-score of the validation set is used as the evaluation metric. If no significant improvement is observed in the metric over 20 consecutive training epochs, the training is terminated, and the best model parameters are saved.
**Algorithm 1:** MGDA-PIAE Training ProcedureInput: Training data X; Max epochs E; Output: Trained model parameters θ; Thresholds $\delta_{\mathrm{recon}}$, $\delta_{\mathrm{phys}}$
**Initialize** encoder/decoder parameters θ
**for**
*epoc*h = 1 **to**
E
**do**:
  Forward pass: $\hat{X}\leftarrow \mathrm{Decoder}(\mathrm{Encoder}(X))$
  Compute losses: $L_{\mathrm{recon}}$ (Equation (6)) and $L_{\mathrm{phys}}$ (Equation (5))
  Compute gradients: $g_{1}\;=\;\nabla_{\theta }L_{\mathrm{recon}}$, $g_{2}\;=\;\nabla_{\theta }L_{\mathrm{phys}}$
  Normalize gradients using Equation (8): $g_{1}^{\prime },{\;g}_{2}^{\prime }$
  Solve Pareto weight γ via Equation (4)
  Joint gradient: $g^{*}\leftarrow \gamma g_{1}^{\prime }\;+\;(1\;-\;\gamma )g_{2}^{\prime }$
  Update: $\theta \leftarrow \theta \;-\;\mathrm{lr}\cdot g^{*}$
**end for**
Compute $\delta_{\mathrm{recon}}$ and $\delta_{\mathrm{phys}}$ via Equations (10) and (11)

### 2.3. Fault Detection Strategy and Model Evaluation

#### 2.3.1. Threshold for Fault Detection Decision

The fault detection framework in this study is based on two adaptive thresholds, and fault judgment is performed through a joint logic.

(1) Two Adaptive Thresholds

Reconstruction error threshold: This is generated by taking 99% of the reconstruction errors of each sensor feature on the training set. It measures the ability of the model to reconstruct each sensor reading.

(10) $$\delta_{\mathrm{recon}}\;=\;\mathrm{Percentile}(\{e_{j}^{\mathrm{train}}\},\;99\%)$$

Physical constraint threshold: Based on the theoretical deviation of the thermal balance equation on the training set, this paper sets an adjustable threshold η, which is an integer between 80% and 99%. It measures whether the system operating state complies with physical laws.

(11) $$\delta_{\mathrm{phys}}\;=\;\mathrm{Percentile}(\{p_{j}^{\mathrm{train}}\},\;\eta )$$

where $\left\{e_{j}^{\mathrm{train}}\right\}$ and $\left\{p_{j}^{\mathrm{train}}\right\}$ denote the set of reconstruction errors and the set of physical residuals for all samples in the training set, respectively.

(2) Fault Judgment Logic

The final fault judgment adopts the logical OR principle: a fault is determined if the error of any channel exceeds its corresponding threshold. This logic ensures comprehensive coverage of different types of faults.

The final fault decision function is given as follows:

(12) $$\mathrm{Fault}(x_{j})\;=\;I[(\overset{7}{\underset{i=1}{⋁}}\left(e_{j}\;>\;\delta_{\mathrm{recon}}\right))\;\vee \;\left(p_{j}\;>\;\delta_{\mathrm{phys}})\right]$$

where I[·] is the indicator function, and ∨ denotes the logical OR operation.

#### 2.3.2. Performance Evaluation Metrics

To comprehensively and scientifically evaluate the fault detection performance of the model and avoid evaluation bias caused by a single metric, this study adopts multiple evaluation indicators to measure the model’s performance in fault identification from different perspectives, ensuring the objectivity and practicality of the evaluation results.

The confusion matrix in Table 2 is the foundation for the performance evaluation of classification models. It is a cross-tabulation between predicted results and actual conditions. In the binary fault detection scenario (normal vs. fault), the structure of the confusion matrix is as follows:

Where TP (True Positive): the number of faults correctly detected; FP (False Positive): the number of normal cases misclassified as faults (i.e., false alarms); FN (False Negative): the number of faults missed by the model (i.e., missed detections); and TN (True Negative): the number of normal cases correctly identified.

Based on the four basic elements in the confusion matrix, the following core evaluation metrics can be calculated [23]:

(1) Precision

Precision measures the proportion of truly faulty samples among all samples predicted as faulty by the model. The calculation formula is given as follows:

(13) $$\mathrm{Precision}\;=\;\frac{\mathrm{TP}}{\mathrm{TP}\;+\;\mathrm{FP}}$$

In the actual operation and maintenance of HVAC systems, frequent false alarms lead to alarm fatigue, causing operation and maintenance personnel to become numb to or even ignore system warnings, thereby delaying the response to real faults.

(2) Recall

Recall reflects the model’s ability to successfully identify faults from all truly faulty samples. The calculation formula is given as follows:

(14) $$\mathrm{Recall}\;=\;\frac{\mathrm{TP}}{\mathrm{TP}\;+\;\mathrm{FN}}$$

A high recall rate means the system has a strong ability to capture real faults, making it a core metric for ensuring the safe and stable operation of HVAC systems.

(3) F1-Score

The F1-score is the harmonic mean of precision and recall, used to comprehensively balance the trade-off between the two metrics. The calculation formula is given as follows:

(15) $$F1\;=\;2\cdot \frac{\mathrm{Precision}\;\times \;\mathrm{Recall}}{\mathrm{Precision}\;+\;\mathrm{Recall}}$$

In HVAC fault detection, a typical imbalanced classification problem, the F1-score considers the effects of both false alarms and missed detections at the same time. It reflects the model’s comprehensive discrimination capability in practical scenarios better than accuracy, and is therefore regarded as one of the core metrics for evaluating the performance of fault detection systems.

## 3. Experimental Setup

### 3.1. Data Description

The data used in this paper are field-measured data from a data center in Guangzhou, covering the period from 1 January 2022 to 31 December 2024. The sampling interval is 10 min, with approximately 157,800 data records.

(1) Dataset Characteristics

The dataset comprises six sensor variables and the compressor power, specifically: evaporator water entering/leaving temperatures (TEI/TEO), condenser water entering/leaving temperatures (TCI/TCO), chilled/cooling water flow rates (FWE/FWC), and compressor power (kW). These variables are all represented as continuous time-series float data, as illustrated in Table 3.

Specifically, the TEO is concentrated around 14 °C, while the TEI is typically 3–5 °C higher than the TEO. The TCI and TCO mostly range between 12 °C and 40 °C, exhibiting a more dispersed distribution. The mean values of the FWE and FWC fall within the range of 2–3 × 10^3^ m^3^/h, with the average FWC being 20–30% higher than the FWE.

(2) Data Pre-processing

Outlier removal: Since our training set needs to reflect a “pure normal” healthy operating state, we employed a statistics-based interquartile range (IQR) method combined with physical extreme value ranges (derived from equipment nameplates and design parameters) to eliminate non-physical extreme points caused by the poor transient anti-interference capability of the sensors.

Data cleaning and missing value processing: Due to occasional communication delays or packet losses during the long-term operation of the sensors, a small number of missing values exist in the dataset. Given the time continuity of the thermodynamic parameters of the chiller, we applied linear interpolation to fill in these short-term missing data.

Data normalization: To eliminate the differences in dimensions and numerical magnitudes among various sensor variables (e.g., temperature, flow rate, and power) and to accelerate the convergence of the deep neural network, we utilized min–max normalization to uniformly scale all features into the [0, 1] interval.

(3) Dataset Partitioning

The dataset is partitioned into three subsets, as detailed in Table 4. The complete data from 2022 and 2023 are allocated as the training set and the validation set, respectively. Both sets exclusively utilize pure normal operational data to ensure that the MGDA-PIAE model can effectively learn the thermodynamic constraints of the system under healthy conditions. The test set is constructed based on the normal operational data from 2024, employing a hybrid fault injection strategy: approximately 26,058 samples from the first half of the year are maintained in a fault-free state, while sensor bias faults are injected into the samples from the second half of the year.

(4) Multi-Level Bias Fault Injection

To comprehensively evaluate the sensitivity of the MGDA-PIAE model to physics-violating faults, bias faults with 20 magnitudes are independently injected into each sensor. A fixed bias is added to the measured values of the target sensors in the second half of the test set, so as to simulate the systematic measurement errors of sensors caused by long-term operation, as shown in Table 5.

### 3.2. Model Parameter Settings

The optimal hyperparameters of the AE model and the MGDA-PIAE model are determined via grid search, and the k-fold cross-validation method (k = 5) is adopted to mitigate the potential overfitting risk. Table 6 shows the hyperparameter optimization ranges and the final parameter settings during the grid search.

The selection of k = 5 is based on the large scale of the training and validation datasets (each exceeding 500,000 samples). Under this data volume, a 5-fold partition already yields validation folds of over 100,000 samples, providing statistically robust performance estimates. Increasing k (e.g., to 10) would substantially raise computational costs during grid search while offering a negligible marginal benefit in variance reduction. Thus, k = 5 represents a practical trade-off between estimation reliability and computational efficiency.

Regarding the initialization strategy for hyperparameters (i.e., the baseline settings at the beginning of training), we followed the conventions of similar deep learning tasks: the initial learning rate was set to 5 × 10^−4^ (to coordinate with the adaptive adjustment mechanism of the Adam optimizer), and the initial search range for the batch size was set to [16, 32, 64, 128]. Subsequently, through grid search and cross-validation, the optimal hyperparameter combination presented in Table 6 was ultimately determined (e.g., an optimal batch size of 32 and a final stable learning rate of 5 × 10^−6^).

## 4. Results and Discussion

To explicitly verify the independent functions and synergistic effects of the core modules in the proposed framework, this section is organized following a systematic ablation study logic. Specifically, Section 4.1 ablates the MGDA optimization module to evaluate the independent contribution of dynamic weight optimization by comparing it with traditional fixed weights. Section 4.2 ablates the physical constraint module by progressively comparing the purely data-driven model (AE), the static-weight physical model (PIAE), and the fully integrated model (MGDA-PIAE), thereby quantifying the independent contribution of physical laws and their synergistic effects with the MGDA module. Subsequent sections further discuss the model’s robustness and adaptability.

### 4.1. Evaluation of the Optimization Module: Comparison of Weights Between MGDA-PIAE and Traditional PIAE

Based on the training configuration with a batch size of 32 and a learning rate of 5 × 10^−4^, this section compares the differences between the MGDA dynamic weights and the traditional PIAE fixed weights from two aspects: weight evolution characteristics and the fixed weight optimization process.

#### 4.1.1. Dynamic Weight Variation in MGDA-PIAE

**Figure 4:**
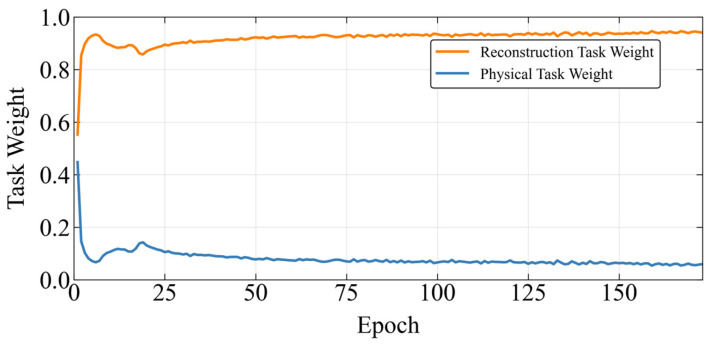
Weight variation during the training process.

**Figure 5:**
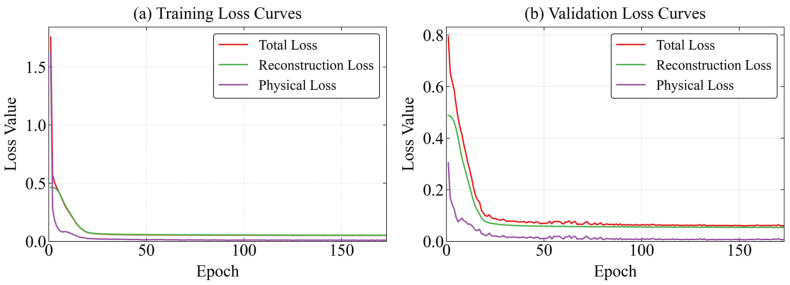
Convergence process of training and validation losses.

The dual-task weights solved by MGDA exhibit a two-stage adaptive evolution that is highly coupled with the model learning process (Figure 4 and Figure 5):

Stage 1 (Epoch 0–20): The rapid dominance stage of data reconstruction.

The weight of the reconstruction task rose rapidly from the initial 0.5 to approximately 0.95, while the weight of the physical task dropped sharply from 0.5 to around 0.05. Corresponding to the loss curves, the reconstruction loss quickly decreased to below 0.05 and stabilized, and the physical loss dropped to below 0.1 synchronously, achieving rapid convergence of the total loss. In this stage, the model prioritizes learning the statistical fitting features of the data, laying a foundation for the subsequent embedding of physical constraints.

Stage 2 (Epoch 20–160): The Pareto optimal stability stage.

The weight of the reconstruction task stabilized in the range of 0.95 ± 0.02, and the weight of the physical task remained within 0.05 ± 0.01 with minimal fluctuation. The reconstruction loss and physical loss of both the training set and validation set remained stable without significant fluctuations. In this stage, MGDA has alleviated the dual-task gradient conflict, and the model parameter update direction is close to the Pareto optimal front. The high proportion of reconstruction weight ensures the accuracy of data fitting, and the low proportion of physical weight constrains the physical rationality of the output, realizing the dynamic balance of the two tasks.

#### 4.1.2. Grid Search for Weight Optimization of Fixed-Weight PIAE

**Figure 6:**
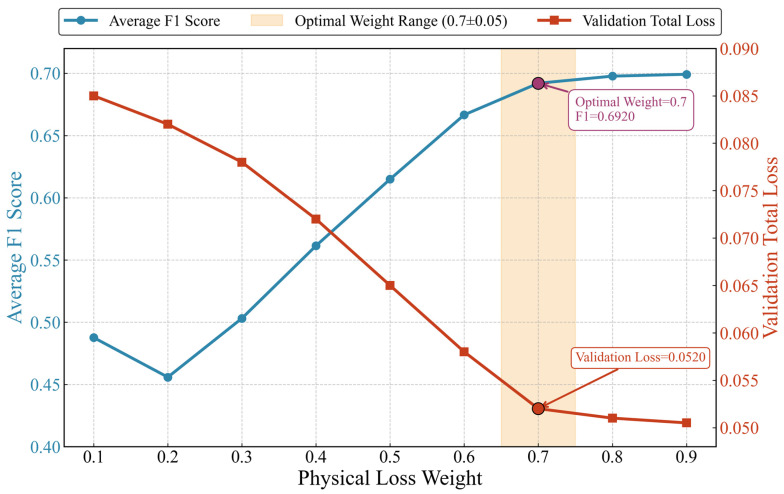
Variation in average F1-score and validation set total loss with physical loss weight.

The traditional PIAE needs to traverse the candidate physical loss weights (γ) through a grid search to determine the optimal value. In this experiment, the candidate weight set γ ∈ [0.1, 0.2, 0.3, 0.4, 0.5, 0.6, 0.7, 0.8, 0.9] was selected with Epoch = 20, and the optimization was performed with the total loss of the validation set and the average F1-score as indicators (Figure 6).

Weight evolution law: With the increase in physical loss weight, the total loss of the validation set showed a monotonous decreasing trend (from 0.085 to 0.0505), and the average F1-score rose rapidly at first and then tended to be flat (the F1-score increased from 0.4876 to 0.692 when the weight increased from 0.1 to 0.7 in the early stage, and only increased slightly to 0.6992 after 0.7). There is a trade-off between the total loss of the validation set and the average F1-score.

Determination of the optimal weight: When γ = 0.7, the average F1-score reached 0.692 (close to the peak), and the total loss of the validation set dropped to 0.052. The weighted balance between reconstruction loss and physical loss achieved a globally optimal state, which not only ensured a significant reduction in the mean value of thermal equilibrium residual and the rationality of physical constraints, but also maximized the ability to distinguish abnormal data. Therefore, γ = 0.7 is the optimal physical weight point of PIAE.

Although the model achieves the optimal trade-off between physical constraint compliance and anomaly detection performance at this weight, the traditional grid search requires manual traversal of candidate weights and can only achieve local optimization based on a limited candidate set, making it difficult to cover the global balance of the continuous weight space.

The main advantages of MGDA dynamic weights compared with traditional PIAE fixed weights are as follows:

(1) Hyperparameter tuning cost: MGDA adaptively determines the dual-task priority through gradient information without manual weight traversal; the traditional PIAE requires grid search for candidate weights, and the tuning cost increases with the complexity of the operating conditions.
(2) Balancing capability: MGDA dynamic weights can adapt to the task requirements of the entire training cycle (fitting data first, then constraining physics) to achieve global Pareto optimality; the traditional PIAE fixed weights can only achieve local balance under a single operating condition with poor cross-scenario adaptability; that is, such local optimal weights do not mean global optimal weights.

It should be noted that the above analysis assumes a strictly pure training set (as described in the pre-processing in Section 3.1). In actual deployment, if a small number of anomalies inadvertently remain in the training data, the MGDA-PIAE model should theoretically exhibit stronger robustness than conventional AE and PIAE models. This is because the physical constraint loss acts as an implicit regularization term: once contaminated samples violate the thermal balance equation, the MGDA mechanism will naturally reduce the reconstruction weight to suppress the learning of spurious patterns. A systematic investigation of model performance under non-ideal training data conditions is reserved for future work.

### 4.2. Role of Physical Constraints and Synergy: Comparative Analysis of MGDA-PIAE and Traditional Models

To verify the performance of the MGDA-PIAE model in fault detection tasks, this section compares its detection effects with those of the traditional Autoencoder (AE) model in different scenarios.

#### 4.2.1. Performance Comparison Under Different Training Batch Sizes

**Figure 7:**
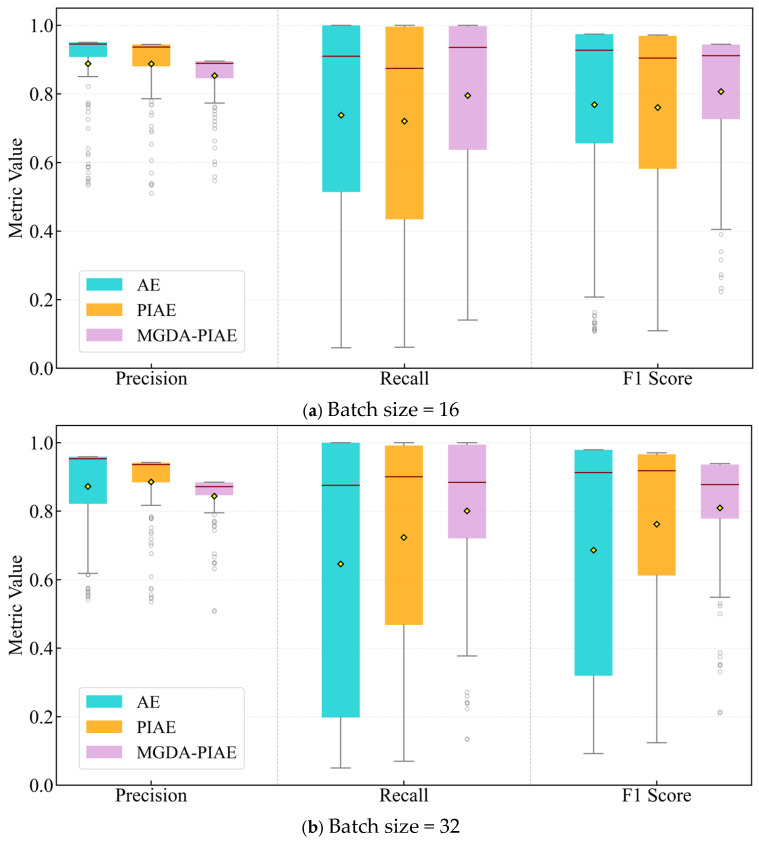

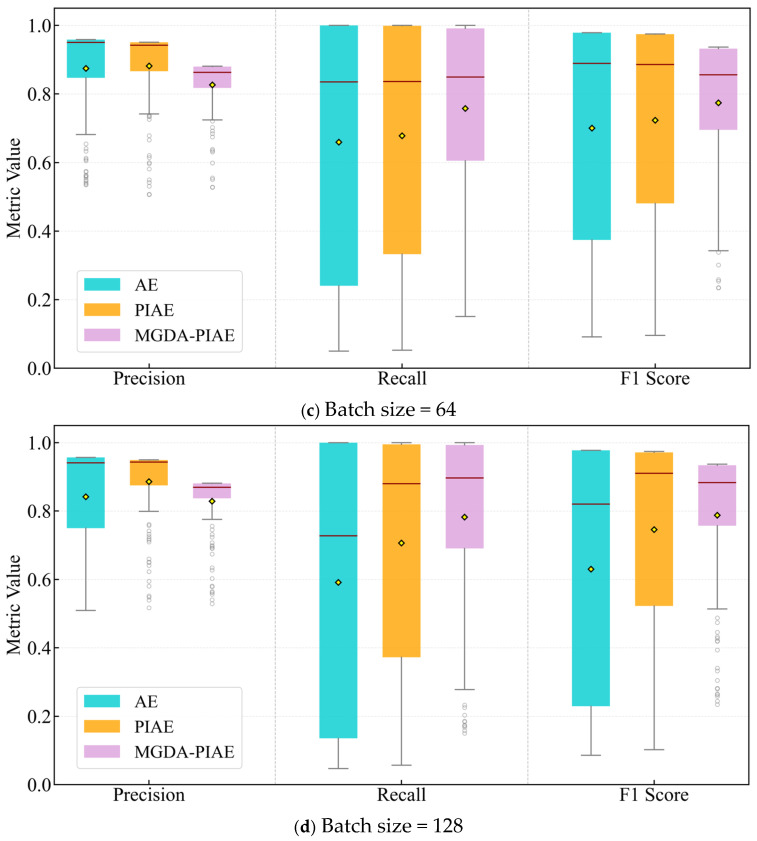
Results of MGDA-PIAE, PIAE and AE under different batch sizes.

Table 7 shows the comparison results under different batch sizes with the optimal hyperparameter combination (learning rate = 5 × 10^−6^, latent space dimension = 64). The fault detection performance of the AE and MGDA-PIAE models is compared and analyzed from three indicators—Precision, Recall and F1-Score—with the box plot distribution shown in Figure 7.

It can be seen from the figures that the boxes of the MGDA-PIAE model are generally shorter than those of PIAE and AE, indicating that the distribution of the middle 50% of fault detection results is more concentrated and the performance fluctuation of the model under different fault grades is smaller, which shows that the MGDA-PIAE has better generalization ability and robustness. The performance of both models changes correspondingly with the increase in batch size.

The average precision of the three models remains above 0.8 under different batch sizes. The MGDA-PIAE model not only maintains a high level of precision, but also significantly improves the recall (an increase of 20% compared with AE and 11% compared with PIAE), resulting in a higher F1-Score, which is 15.9% and 8.1% higher than that of AE and PIAE, respectively. This indicates that the MGDA-PIAE achieves a better balance between accuracy and comprehensiveness.

The MGDA-PIAE achieves the best performance when the batch size is 32, with its recall increased by 25% and F1-Score by 19% compared with the AE model; compared with PIAE, the recall is increased by 11.1% and the F1-Score by 6.5%.

**Figure 8:**
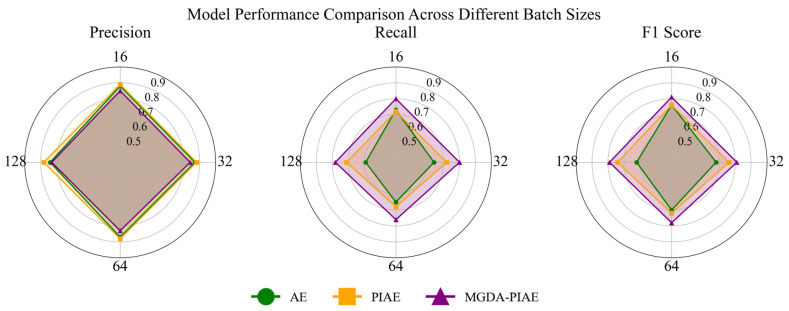
Average detection performance of MGDA-PIAE, PIAE and AE under different batches.

Specifically, as shown in Figure 8, in the Precision radar chart, AE and PIAE are slightly superior to the MGDA-PIAE model, while in the Recall and F1-Score charts, the areas enclosed by AE and PIAE are completely covered by the MGDA-PIAE model, which means that the fault detection effect of the proposed model is better than that of the traditional models.

In the wide range of batch size from 16 to 128, the recall of the MGDA-PIAE model is stably higher than that of the AE and PIAE models. The improvement in recall leads to a synchronous rise in the F1-Score, indicating that physical constraints effectively guide the model to learn more discriminative features. At the same time, the performance fluctuation of MGDA-PIAE in this interval is small, showing that it has stronger adaptability to hyperparameter changes and a more robust model training process.

When the Batch Size increases to 128, the performance of both models declines, and the advantage of MGDA-PIAE narrows. This indicates that an excessively large batch may affect the optimization effect of physical constraint loss, revealing the applicable boundary of the method.

The MGDA-PIAE model can capture faults more comprehensively while maintaining a high level of precision, achieving the optimal balance between detection performance and robustness. Although the performance slightly declines with the increase in batch size, the MGDA-PIAE maintains a stable advantage under all settings.

#### 4.2.2. Analysis of Sensor Type and Fault Degree

The MGDA-PIAE model has a significant improvement in overall performance compared with PIAE and AE models, especially in the detection results of most sensors and fault degrees. In this section, the training batch size is fixed at 32 to analyze the F1-Score performance of MGDA-PIAE, PIAE and AE for six sensors (FWC, FWE, TCI, TCO, TEI, TEO) under 20 gradual fault degrees.

**Figure 9:**
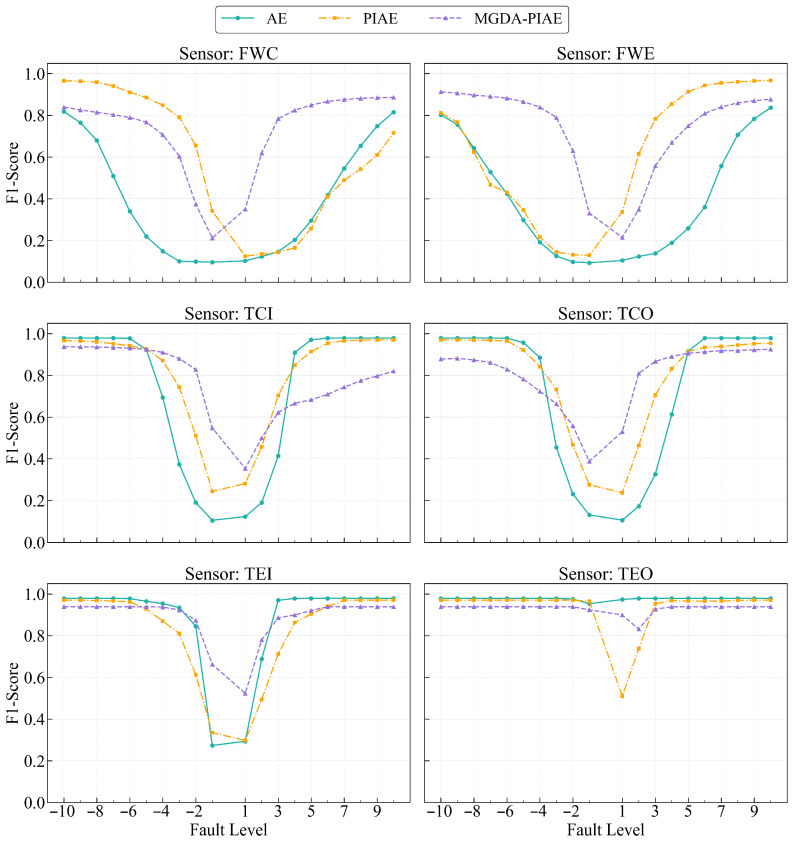
F1-Score comparison of AE, PIAE and MGDA-PIAE models (by sensor).

**Figure 10:**
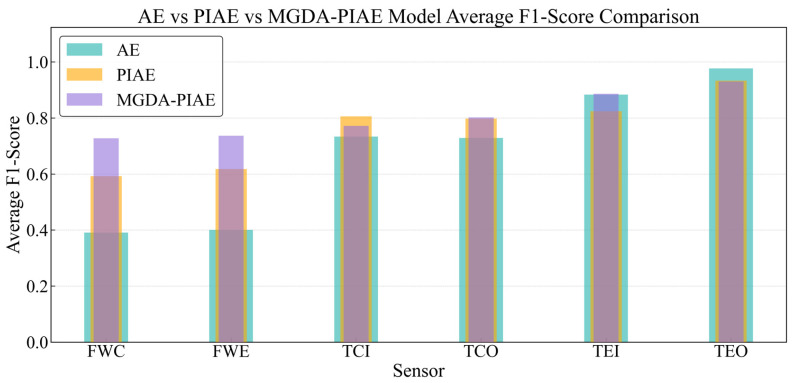
Comparison of average F1-Score of AE, PIAE and MGDA-PIAE models for different sensors.

Figure 9 shows the F1-Score detection results of the three models for six sensors under 20 fault degrees, and Figure 10 is a bar chart of the average F1-Score of the three models for different sensors.

Compared with AE, the MGDA-PIAE model has the best improvement effect on FWC and FWE sensors, and its overall performance curve is above that of AE. Specifically, the MGDA-PIAE model can maintain an F1-Score of about 0.8 when the deviation grade of FWC is −6 and 4 (and above), and the deviation degree of FWE is −3 (and above), while the F1-Score of AE drops to 0.1~0.4 at the same grade. From the average values in Figure 10, the F1-Scores of MGDA-PIAE for FWC and FWE sensors (0.7280, 0.7370) are increased by more than 80% compared with AE (0.3910, 0.4006).

In comparison with PIAE, the MGDA-PIAE has a significant overall performance improvement in the flow sensors. It can be seen from Figure 10 that the average values of traditional PIAE for FWC and FWE are 0.5952 and 0.6180, respectively, with an increase of about 20%. Specifically, on the negative deviation fault grades of the FWC sensor, although the effect of the traditional PIAE is better than that of the MGDA-PIAE, the leading margin is small and the F1-Score of the MGDA-PIAE is more stable with smaller fluctuations; on the positive deviation grade faults, the MGDA-PIAE is significantly ahead. For the FWE sensor, the advantages and disadvantages of the MGDA-PIAE model in the positive and negative deviations are the opposite to the above results. This indicates that the MGDA optimization strategy effectively alleviates the performance imbalance problem of the single-weight PIAE model under specific fault modes.

For temperature sensors, Figure 10 shows that the average F1-Scores of the three models have little difference. The average F1-Scores of MGDA-PIAE, AE and PIAE for the main temperature sensors (TCI, TCO, TEI) are extremely close, but they show complementary advantages at different fault grades. For example, at some extreme negative deviation grades (such as −1, 1) of the TCI sensor, the F1-Scores of AE and PIAE are slightly higher than those of MGDA-PIAE; while at some positive deviation grades (such as 6, 7), the MGDA-PIAE is slightly superior. This reflects that the performance difference between the two models with physical constraints is not as significant as that on flow sensors in temperature parameter fault detection, but the overall performance of MGDA-PIAE is smoother.

**Figure 11:**
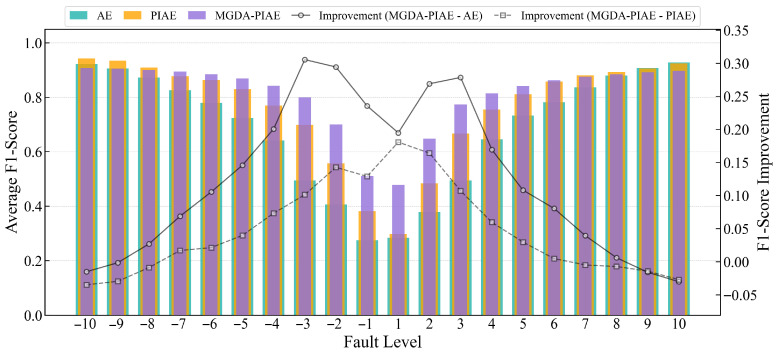
Comparison of average F1-Score and improvement degree of AE, PIAE and MGDA-PIAE models at different fault grades.

Figure 11 shows the average F1-Scores of MGDA-PIAE, PIAE and AE at different fault grades. On the whole, the MGDA-PIAE has achieved a significant improvement in fault detection performance compared with PIAE and AE models, and its average F1-Score is better than that of PIAE and AE.

At high fault degrees (such as ±10, ±9, ±8), the MGDA-PIAE is sometimes slightly lower than PIAE and AE, and sometimes the average F1-Scores of the three models are almost the same. The improvement degree (blue line) is stably at about 0.1, and the difference in fault detection effects between the models is small.

With the gradual decrease in the fault degree, the improvement degree of MGDA-PIAE compared with PIAE and AE is comprehensively higher. From ±7 to ±3, the performance advantage of MGDA-PIAE is gradually highlighted: the gap in the average F1-Score between MGDA-PIAE and the other two models continues to expand, and the improvement degree rises synchronously; the improvement degree reaches a peak of 0.25~0.3 near the fault grade of −4. When the fault degree drops to within ±2, the gap in the average F1-Score between MGDA-PIAE and PIAE/AE gradually narrows, but the MGDA-PIAE still performs the best.

In summary, the MGDA-PIAE model effectively balances physical constraints and reconstruction accuracy through the multiple gradient descent algorithm, and achieves the best comprehensive performance in fault detection tasks. Its detection accuracy on key flow sensors (FWC, FWE) is significantly higher than that of AE and PIAE, it has comparable performance with the two models on temperature sensors, and it exhibits better stability and robustness at different fault grades, especially in moderate deviation faults.

### 4.3. Scenario Analysis of Physical Threshold Adaptability for MGDA-PIAE

The requirements for the fault detection accuracy of HVAC systems vary in different application scenarios. For example, in data centers, air conditioning systems need to operate 24 h a day all year round. Excessive false fault alarms may cause alarm fatigue among operation and maintenance personnel, leading them to ignore system warnings and thus delaying the handling of real faults. In addition, high operation and maintenance costs and ineffective maintenance will bring additional losses. Therefore, in this case, the false alarm rate needs to be controlled as a priority. In environments such as cleanrooms, the requirements for temperature, humidity and cleanliness are very strict. Missed detection of sensor faults may lead to unqualified products and thus greater economic losses. Therefore, the missed alarm rate should be ensured to be low in such scenarios.

In this section on the premise of fixing the reconstruction error threshold at 99%, the physical constraint threshold η is set to an integer interval from 80% to 99%, and systematic tests are carried out on samples of six sensors and 20 fault degrees to analyze the influence law of the physical constraint threshold on detection performance and evaluate the adaptability and robustness of the model in different fault scenarios. Table 8 shows the average detection results of the MGDA-PIAE model under different thresholds.

#### 4.3.1. Threshold Adaptation for the “Low False Alarm” Scenario

Precision reflects the model’s ability to control the false detection of normal samples, which is a priority evaluation index for stability-sensitive scenarios such as data centers and ICUs in the field of fault detection.

**Figure 12:**
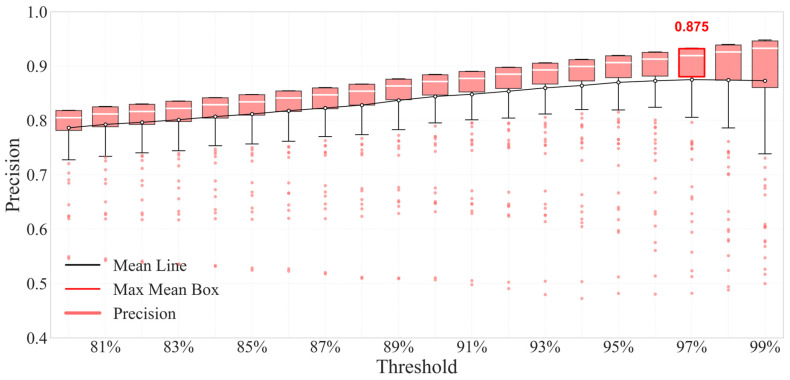
Box plot of precision distribution under different physical thresholds.

It can be seen from Figure 12 that with the increase in the physical constraint threshold η, the mean line of precision (black line) shows a monotonous rising trend; when η = 97%, the corresponding box is the high-value box marked by the red frame, whose mean line reaches 0.875, the upper edge of the box is close to 0.95 and the lower edge is stable in the range above 0.8.

Combined with the data in the table, the quantitative value of Precision at this threshold is 0.8750, which is a relatively high level in the whole threshold interval. From the perspective of sample robustness, the scatter points (corresponding to different sensors and fault degrees) at this threshold are concentrated in the range of 0.6~0.9 without large fluctuations. It shows that near η = 97%, the model can maintain high precision for samples of different types of sensors and different fault degrees with less false alarm interference, which is in line with the requirements of scenarios requiring “low false alarm,” such as data centers and ICUs.

#### 4.3.2. Threshold Adaptation for the “Low Missed Alarm” Scenario

Recall reflects the model’s ability to control the missed detection of fault samples, and the missed alarm rate is prioritized to be controlled in environments such as cleanrooms, pharmaceutical workshops and food production workshops.

**Figure 13:**
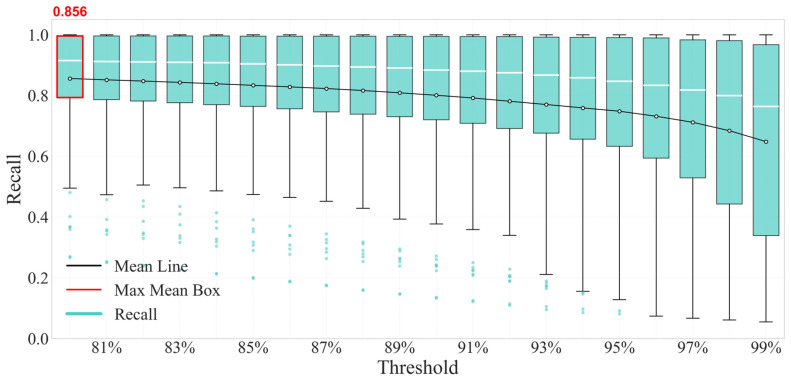
Box plot of recall distribution under different physical thresholds.

It can be seen from Figure 13 that when the physical constraint threshold η = 80%, the corresponding box is the highest mean box marked by the red frame, whose mean line (black line) reaches 0.8559, the upper edge of the box reaches 1.0 and the lower edge is in the range above 0.5.

Combined with the data in the table, the quantitative value of recall at this threshold is 0.8559, which is the maximum value in the whole threshold interval. From the perspective of sample coverage, although the scatter points corresponding to this threshold in the figure have a certain distribution, they are generally concentrated within the box without a large number of significantly deviated abnormal points. It shows that near η = 80%, the model can maintain a high recall for samples of six sensors and 20 fault degrees with low missed detection risk, which is suitable for the requirements of scenarios requiring “low missed alarm” such as pharmaceutical and food production workshops.

#### 4.3.3. Threshold Allocation for Other Scenarios

The F1-Score is the harmonic mean of Precision and Recall, reflecting the model’s ability to balance the costs of “false alarm and missed alarm”.

**Figure 14:**
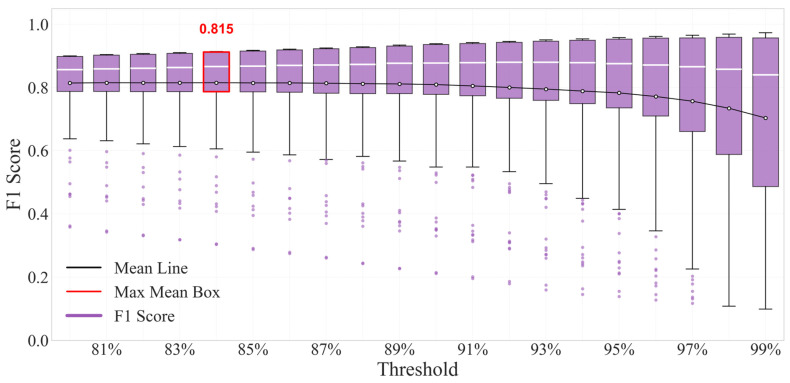
Box plot of F1-Score distribution under different physical thresholds.

It can be seen from Figure 14 that when the physical constraint threshold is in the range of 80%~84%, the mean line of F1-Score (black line) remains stable above 0.81; among them, the box corresponding to η = 84% is the high-value box marked by the red frame, whose mean line reaches 0.815, and the upper and lower edges of the box are in the range of 0.9 and 0.6, respectively.

The MGDA-PIAE model can achieve precise adaptation to three types of HVAC scenarios—“low missed alarm”, “low false alarm” and “balanced performance”—by adjusting the physical constraint threshold. At the same time, the model also has good performance on samples with multiple sensors and multiple fault degrees under the corresponding thresholds.

## 5. Conclusions

This study proposes a Multi-Gradient-Descent-Integrated Physics-Informed Autoencoder (MGDA-PIAE) for sensor fault detection in data center chillers. By dynamically balancing data reconstruction accuracy and physical consistency through the Multiple Gradient Descent Algorithm (MGDA), the model effectively addresses the gradient conflict that is inherent in traditional physics-informed models. Validated with real-world operational data from a data center in Guangzhou, the main conclusions are as follows:

(1) The MGDA-PIAE model achieves a superior balance between reconstruction accuracy and physical plausibility. Compared to traditional AE and PIAE models, it increases the average recall and F1-score by approximately 20% and 10%, respectively, while maintaining high precision.
(2) In the detection of critical flow sensors, the model shows a significant performance leap, with the F1-score increasing by more than 80% compared to AE and about 20% compared to PIAE. Notably, in the moderate deviation fault range (±3 to ±7), the performance improvement peaks at 25–30%, demonstrating exceptional fault feature discrimination and generalization.
(3) By adjusting a single physical constraint threshold, the model can flexibly switch between “high precision” (precision of 0.875 at 97% threshold), “high recall” (recall of 0.856 at 80% threshold), and “balanced performance” (F1-score of 0.815 at 84% threshold). This allows for precise adaptation to diverse maintenance scenarios with varying fault tolerance requirements, such as data centers and cleanrooms.

However, this study has certain limitations. The current model is trained and evaluated primarily on clean datasets, without analyzing its response to mixed or anomalous data. It focuses on sensor bias faults and does not yet address more complex fault types, such as drift, stuck, or precision attenuation, which may limit the adaptability to diverse chiller sensor faults in real-world data centers. Furthermore, the physical constraint model considers only the chiller itself and does not integrate auxiliary refrigeration equipment such as cooling towers and pumps, preventing a fully system-level fault detection. Finally, model training and validation are based solely on the mechanical cooling mode, while free cooling and hybrid modes, which involve different operating parameters and thermal balances, remain untested and require further verification.

## Figures and Tables

**Table 1:** Differences between MGDA-PIAE and traditional PIAE.

Comparison Dimension	Traditional PIAE	MGDA-PIAE Fusion Method
Weight Adjustment	Fixed weight or adaptive parameter adjustment	Dynamic weight optimization
Physical Constraint	Usually no physical constraint	Physical information enhancement
Parameter Sharing	Task-independent or limited sharing	Dual-task shared architecture
Objective Conflict Handling	Weighted sum, prone to imbalance	Pareto optimization balance

**Table 2:** The confusion matrix.

	True Positive (Fault)	True Negative (Normal)
Predicted Fault	True Positive (TP)	False Positive (FP)
Predicted Normal	False Negative (FN)	True Negative (TN)

**Table 3:** Sensor data inventory of chillers in a Guangzhou data center.

Sensors	Descriptions	Units
TEI	Temperature of Evaporator Water In	°C
TEO	Temperature of Evaporator Water Out	°C
TCI	Temperature of Condenser Water In	°C
TCO	Temperature of Condenser Water Out	°C
FWE	Flow Rate of Evaporator Water	m^3^/h
FWC	Flow Rate of Condenser Water	m^3^/h

**Table 4:** Dataset partitioning.

Dataset Category	Data Period	Data Characteristics
Training Set	2022	Normal data
Validation Set	2023	Normal data
Test Set	2024	Approximately 26,058 samples in the first half of the year remained fault-free, while in the second half, sensor bias faults were injected into the samples

**Table 5:** Sensor fault bias levels.

Sensor Type	Fault Parameter Range	Step Interval	Fault Levels
Temperature sensors (TEI/TEO/TCI/TCO)	−5.0 °C ~ +5.0 °C	0.5 °C	20
Flow sensors (FWE/FWC)	−20% ~ +20%	2%	20

**Table 6:** Model hyperparameter configuration.

Hyperparameter	Optimization Range	Optimal Value
Number of hidden layers (Encoder/Decoder)	[2–4]	3/3
Number of neurons	[32–128; 16–64; 8–32]	[128; 64; 32] (Encoder) [32; 64; 128] (Decoder)
Initial learning rate	[5 × 10^−6^, 5 × 10^−4^]	5 × 10^−6^
Activation function	‘sigmoid’, ‘tanh’, ‘relu’	‘relu’
Optimizer	Adam, SGD, RMSprop	Adam
Batch size	[16, 32, 64, 128]	32
Training epochs	[50, 200]	160 (early stopping triggered)
Threshold setting	—	Reconstruction error: 99th percentile Physical constraint: 80%–99th percentile (adjustable)

**Table 7:** Performance comparison of MGDA-PIAE, AE, and PIAE across batch sizes.

Batch Size	Model	Precision	Recall	F1-Score
Average	AE	0.86	0.65	0.69
	PIAE	0.88	0.70	0.74
	MGDA-PIAE	0.84	0.78	0.80
16	AE	0.88	0.73	0.76
	PIAE	0.89	0.72	0.76
	MGDA-PIAE	0.85	0.80	0.81
32	AE	0.87	0.64	0.68
	PIAE	0.88	0.72	0.76
	MGDA-PIAE	0.84	0.80	0.81
64	AE	0.87	0.65	0.70
	PIAE	0.88	0.68	0.72
	MGDA-PIAE	0.83	0.76	0.78
128	AE	0.84	0.59	0.62
	PIAE	0.88	0.71	0.74
	MGDA-PIAE	0.83	0.78	0.79

Note: The values in the table are the average values of six sensors under 20 fault degrees in this scenario.

**Table 8:** Comparison of detection indicators of the MGDA-PIAE model under different physical thresholds.

Physical Constraint Threshold (%)	Precision	Recall	F1-Score
80	0.7863	0.8559	0.8143
81	0.7926	0.8515	0.8152
82	0.7964	0.8474	0.8149
83	0.8012	0.8431	0.8149
84	0.8070	0.8385	0.8152
85	0.8117	0.8334	0.8146
86	0.8178	0.8284	0.8146
87	0.8227	0.8228	0.8136
88	0.8282	0.8164	0.8122
89	0.8368	0.8088	0.8113
90	0.8440	0.8007	0.8092
91	0.8481	0.7918	0.8051
92	0.8537	0.7810	0.8002
93	0.8598	0.7702	0.7951
94	0.8642	0.7591	0.7888
95	0.8700	0.7481	0.7829
96	0.8728	0.7317	0.7715
97	0.8750	0.7117	0.7568
98	0.8742	0.6840	0.7343
99	0.8730	0.6482	0.7037

## Data Availability

The authors do not have permission to share data.
